# A multi-phenotypic imaging screen to identify bacterial effectors by exogenous expression in a HeLa cell line

**DOI:** 10.1038/sdata.2018.81

**Published:** 2018-05-15

**Authors:** Adam Collins, Alan Huett

**Affiliations:** 1 School of Life Sciences, University of Nottingham, Queens Medical Centre, Nottingham NG7 2UH, UK

**Keywords:** High-throughput screening, Fluorescence imaging, Pathogens, Macroautophagy

## Abstract

We present a high-content screen (HCS) for the simultaneous analysis of multiple phenotypes in HeLa cells expressing an autophagy reporter (mcherry-LC3) and one of 224 GFP-fused proteins from the Crohn’s Disease (CD)-associated bacterium, Adherent Invasive *E. coli* (AIEC) strain LF82. Using automated confocal microscopy and image analysis (CellProfiler), we localised GFP fusions within cells, and monitored their effects upon autophagy (an important innate cellular defence mechanism), cellular and nuclear morphology, and the actin cytoskeleton. This data will provide an atlas for the localisation of 224 AIEC proteins within human cells, as well as a dataset to analyse their effects upon many aspects of host cell morphology. We also describe an open-source, automated, image-analysis workflow to identify bacterial effectors and their roles via the perturbations induced in reporter cell lines when candidate effectors are exogenously expressed.

## Background & Summary

Bacteria secrete a panoply of effector proteins to manipulate potential host cells, mediating invasion or attachment, immune evasion and nutrition. In particular, intracellular bacteria often invade non-phagocytic cells, and then maintain an intracellular niche as they grow and replicate^1^. The best studied of these effector secretion pathways are the Type III and IV secretion systems, which operate as molecular syringes to transfer effector proteins from bacterial cells to target cells^2^. More recently, the type VI secretion system has been described as performing a similar role in some pathogens, as well as mediating inter-bacterial killing and competition^3^.

Despite some recent advances in the identification of secretion signals and motifs^4–7^, there remain very few sequence features that enable robust computational prediction of bacterial effector proteins. This has led to a need to screen for proteins via their effects upon model hosts. Initially such screens were performed using libraries of bacterial mutants^8,9^, but this often fails to find all secreted proteins due to trans-complementation between mutants within a pool, or effector redundancy. More direct approaches such as expression of putative effector proteins in yeast have been able to find new effectors, place their effects within host pathways^10,11^, and more recently demonstrate the synergy and interactions between effectors^12^. In some cases these observations have been extended to other infection models or mammalian cell systems^13^. However, in yeast the primary readout is growth – whilst this makes screening robust and cheap, it gives little additional information on which cellular pathways are involved in effector action, although recently this has been improved with subcellular localisation screening of known secreted effectors in yeast^14^. Extending this approach to mammalian cells, we are able to gain more information by using reporters in pathways of interest, as well as simultaneously imaging a number of cellular components and cell properties. Using fluorescently-tagged bacterial proteins also shows the localisation of specific bacterial proteins within the host cells, offering further insight into their roles and functions. A similar approach has been recently used for assaying human gene function and has been termed cell painting^15^.

Adherent Invasive *Escherichia coli* is a bacterium associated with Crohn’s disease, detected much more frequently within the mucosa of CD patients than healthy controls, and capable of initiating uncontrolled inflammation following other insults in mouse models^16–18^. Unlike many pathogenic *E. coli*, it lacks an identified type III secretion system, but possesses two type VI systems of unknown function. In this study we aimed to find novel effector proteins from AIEC by screening 224 proteins directly in human cells, by transient transfection. The resulting perturbations would be monitored and analysed by automated confocal microscopy of stained cells, and computational image analysis. Since AIEC has a somewhat unique intracellular lifestyle, distinct from other *E. coli* isolates^19^, each bacterial coding sequence cloned was selected based upon its absence from other known *E. coli* pathovars. It is worth noting that the majority of bacterial effector screens have used proteins known or suspected to be Type III, IV or VI secreted effectors; since AIEC lacks Type III or IV secretion, we chose to look for proteins absent from other pathovars as the best proxy for those likely to be associated with the unusual intracellular AIEC lifestyle.

Links between AIEC and CD, and the known role for autophagy in CD pathogenesis, led us to select autophagy as our primary screen readout. We used a HeLa cell line expressing an autophagy reporter, mCherry-LC3 as a model epithelial cell.

To avoid the use of multiple restriction enzyme pairs or proprietary recombination mixes and associated costs, we cloned each bacterial sequence using a restriction-free, recombinase-based system, Seamless Ligation in Cloning Extract^20^. CDSs were cloned downstream of a monomeric, enhanced-brightness GFP variant, mEmerald, and under the control of a CMV promoter to drive high levels of expression. This strategy enabled visualisation of protein localisation. In yeast, GFP fusions have been shown to enhance effector phenotypes, probably by increasing protein stability^10^. In large studies performed to date, the presence of GFP has not been shown to influence the localisation of the majority of proteins tested in human cell lines^21^.

**Figure 1:**
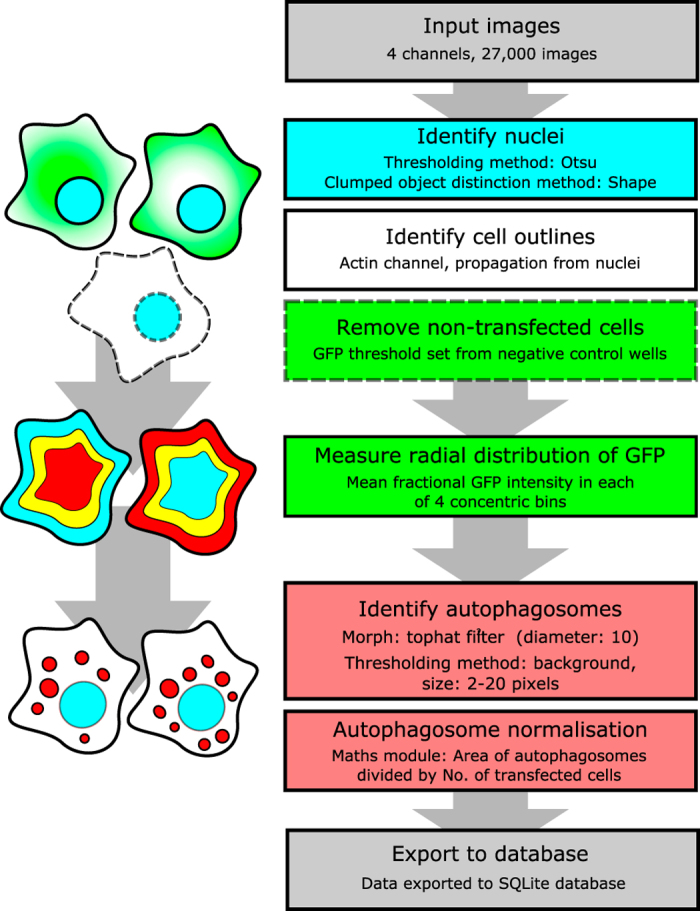
An overview of the image analysis pipeline and the major cellular features measured. A schematic showing the segmentation of cellular features is shown on the left, corresponding to the steps in the analysis pipeline shown in the right workflow panel. First nuclei are identified, then cell outlines. Non-transfected cells (white) are masked (dotted lines), then the subcellular distribution of GFP quantified in radial zones from nuclear centre to cell edges, as indicated by the mock heatmaps. Finally, autophagosomes (red dots) are identified and measured.

mCherry-LC3 HeLa cells were seeded in 96-well plates and transiently transfected with the fusion protein library. After 24 h, plates were fixed and stained for DNA and actin, before being imaged by automated confocal microscopy. Acquired images were then processed by an automated analysis pipeline using CellProfiler^22^ as outlined in Fig. 1. Transfection controls included empty mEmerald vector (expressing mEmerald only), GFP-p62 (known to be an autophagy substrate, inducer of aggregates, and recruiter of the autophagy machinery), and untransfected cells. From this analysis we were able to obtain information about bacterial protein localisation, autophagy status of the transfected cells, nuclear and cellular morphologies, co-localisation of GFP and actin, co-localisation of GFP and autophagosomes and other cellular readouts (Table 1).

This data represents the first examination of eukaryotic localisation and function of the putative pathovar-unique proteins of AIEC LF82. We identify proteins that locate to specific cell areas, both cytoplasmic and nuclear. In particular the nuclear-localising proteins may be of great interest, since they may represent novel factors to manipulate host gene expression, or may themselves be unannotated bacterial transcription factors. These nuclear-localising proteins also present a number of distinct morphologies, possibly related to their functions or sub-nuclear tropisms and association with intra-organelle structures.

We would like to encourage re-use and re-analysis of this dataset by others in the field, either to examine the effects of proteins of interest, or to select potential protein candidates for further studies. Similarly, we believe there are likely to be other phenotypes of interest that we have not analysed, that may be revealed by re-analysis of the images. We also show that screening bacterial proteins in human cell lines is a suitable method to discover both protein localisation (and gain insight into function) and the effects on host cellular morphology and function. Our supplied workflow and image analysis pipeline provide a guide and starting point for other investigators wishing to adopt this approach.

## Methods

### Production of AIEC CDS library

The genome of Adherent Invasive *E. coli* strain LF82 was compared with six other *E. coli* strains (see Table 1) using InParanoid 4.1 (ref. 23). Coding sequences (CDSs) identified as inparalogs between *E. coli* strains were removed from a list of LF82 CDSs. This, alongside data from the LF82 genome annotation and analysis^24^, was used to create a list of LF82 CDSs of interest due to their absence from the comparison strains (Data Citation 1). The genes finally selected had an apparent non-uniform distribution, with many clustering within likely horizontally-transferred DNA regions, zones of genomic plasticity or putative phage integrations. In many cases these were also marked by abrupt alterations in GC content, indicative of recent acquisition. All of these factors confirmed their status as non-core to the *E. coli* genome, and thus likely to be of interest in AIEC specialisation and lifestyle. The resulting gene list had a substantial overlap with that generated by Miquel *et al*.^24^. In some places Miquel *et al* had identified a region of plasticity or pathogenicity island, and we had selected only some of the genes from such a region – we chose to include neighbouring ORFs to complete coverage of the island (with island extent guided by GC content or other markers such as phage genes or tRNAs). As a result of this strategy it is worth noting that the majority of type VI secretion system genes, the chaperone *htrA*, fimbrial subunits, *ibeA* and *ompA/C* are not present in the library, due to the presence of closely related genes in the strains used for comparison. Many of these have been proposed as having virulence roles^25^, mostly in bacterial attachment. Since our screen was for intracellular roles for bacterial proteins, those involved in attachment or not thought to be secreted were not added to the library where the initial orthologue strategy had resulted in their omission. We also cloned 48 genes from LF82 that were present in other *E. coli* to serve as a background set. Our rationale was that should a large proportion of the LF82-restricted library have strong phenotypes when expressed in cells, we would need a control set to compare to. However, in the final analysis we observed no differences in these gene sets. The 48 non-LF82-restricted genes are found in Set 3 part 1 and 2, and sequences are given as a separate FASTA file in Data Citation 1.

Oligonucleotides were designed to amplify and extend each of the CDSs identified, keeping the endogenous stop codon intact, and replacing variant start codons with ATG. Forward and reverse primers also each incorporated 18 bp extensions for subsequent second-round PCR using a pair of common primers. Amplification of CDSs was confirmed via gel electrophoresis. Amplified PCR products were purified using the QIAquick 96 PCR purification kit (QIAGEN), before being used in a second round of PCR. The second PCR added 40 bp extensions matching the sequence flanking the *Eco*RV cut site of the destination vector. The resulting 50 bp matches to the recombination site allow efficient directional recombination cloning, and the second PCR significantly reduced the costs of oligo synthesis by keeping unique oligo pairs below 60 bp. All oligo sequences are available at Harvard Dataverse Depository (Data Citation 2).

Second-round PCR product was utilised in a Seamless Ligation in Cell Extract (SLiCE) reaction^20^ for each CDS; reactions consisted of 100 ng *Eco*RV-HF (NEB) digested pCMV-mEm-4GS vector, 1 μL PPY extract, 1.2 μL 10x SLiCE buffer and 2 μL purified PCR product, made up to 10 μL with water. After incubating at 37 °C for 1 h, SLiCE product was used to transform XL1-Blue *E. coli*, before selecting for successful transformants on LB-agar containing ampicillin. Successfully transformed XL1-Blue were confirmed by colony PCR, grown overnight in deep-well blocks, and subjected to the NucleoBond 96 Xtra HF midiprep kit (Macherey-Nagel). DNA was quantified spectrophotometrically and normalised for concentration prior to Sanger sequencing and transfection. All plasmid constructs used in this screen have been deposited at Addgene and are available for reuse.

### Transfection and screening

HeLa-mCherry-LC3 cells were generated by lentiviral transduction of HeLa cells (line CCL2, ATCC) and cloned by dilution to generate a homogenous population of stable reporter cells. The expanded clone was frozen as stocks in liquid nitrogen and vials thawed one week prior to screening. HeLa-mcherry-LC3 cells were grown at 37 °C, 5% CO_2_ in 10 cm tissue culture dishes using DMEM (high glucose, GlutaMAX, and pyruvate (Gibco)) with 10% iron-supplemented calf serum (Thermo) and 20 μg mL^−1^ gentamicin, and were subdivided upon reaching confluence. Immediately prior to transfection cell media was removed before washing with PBS (without MgCl_2_ and CaCl_2_) (Sigma-Aldrich) and treating with 500 μL trypsin-EDTA (PAA). Cells were collected in complete DMEM without gentamycin, enumerated and diluted to 2.5×10^5^ cells mL^−1^.

Reverse transfection was performed by dispensing 125 ng of each plasmid into wells of a 96-well, black, clear-bottom CELLSTAR Bio-One tissue culture plate (Greiner). 25 μL of Opti-MEM (Gibco) and 0.8 μL of Lipofectamine P3000 reagent (Invitrogen) were mixed, added to each well and incubated for 3 min at 37 °C, 5% CO_2_. 30 μL of Opti-MEM and 0.3 μL of Lipofectamine 3000 reagent (Invitrogen) were then added to each well, and returned to the incubator for 10 min. Finally 100 μL of HeLa-mCherry-LC3 cell suspension was dispensed into each well, and plates incubated for 4 h at 37 °C, 5% CO2, after which medium was replaced with gentamicin-free complete DMEM for overnight incubation. Each plate also contained control wells transfected with GFP-p62 (as a positive control for cytoplasmic localisation and formation of LC3 punta), empty pCMV-mEm-4GS vector, and no-DNA controls. Some untransfected wells were also treated for 3 h with 200 nM rapamycin (Sigma), 50 mM ammonium chloride (Sigma), or both. Cells treated with Torin were exposed to 250 or 500 nM Torin for 3 hours, control cells were exposed to 200 nM rapamycin or DMSO vehicle alone.

Transfected HeLa cells were fixed after 24 hours by removing media and adding 100 μL of 4% formaldehyde in PBS per well. After 15 minutes, wells were washed with PBS, and PBS containing 0.1% Triton X-100 (BDH) applied to each for 5 min to permeabilise cells. Cells were washed twice with PBS, and 100 μL PBS containing 1.5 nM Hoechst 33342 (Molecular Probes) and 13.2 nM phalloidin 647 (Molecular Probes) applied. Plates were incubated for 15 min, the staining solution was removed and cells washed in PBS. Finally, cells were left to rest in 100 μL PBS at 4 °C until imaged.

### High-content image acquisition

Images were acquired with a true point scanning confocal ImageXpress Ultra microscope (Molecular Devices) with a Nikon 40× (NA=0.95) air lens using four fluorescence channels at 405 nm, 488 nm, 561 nm and 635 nm excitation wavelengths. Camera binning was set to 2×2, giving a nominal pixel size of 0.4 μm. All laser power and other imaging settings were retained throughout the screen to minimise differences between imaging batches. The one exception were the Torin validation plates which were captured after laser replacement had taken place. Therefore the absolute intensity values of these, although captured with the same settings, are not as closely aligned as the rest of the data set.

Six non-overlapping fields of view (arranged in a 2×3 grid) were acquired per well, with sites containing 100–150 cells each. To avoid inconsistencies in image acquisition in the outer wells of the 96-well plates, only the centre 60 wells of each plate were used in this screen. Images were acquired in.tiff format, and were used directly for analysis in CellProfiler.

The screen was performed in two replicates, with replicate transfections, staining and imaging performed on separate days.

### High-content analysis

The CellProfiler pipeline initially identifies successfully transfected cells using the Hoechst, actin, and GFP channels. The Hoechst channel is analysed for objects between the ranges of 30–150 pixels, using a Global thresholding strategy and an Otsu thresholding method. The method for distinguishing between objects was shape, and intensity was used to draw dividing lines between clumped objects. Once identified, nuclei were used as seeds from which to propagate for the detection of cell outlines in the actin channel, using an adaptive thresholding strategy and an Otsu thresholding method. Once cells were identified the intensity of GFP within each cell was then determined, and cells with a mean intensity below 0.0245, or a median absolute deviation intensity (MADIntensity) below 0.0013, were considered ‘untransfected’. These untransfected cells are removed from subsequent analysis by generating a mask based upon their actin-derived outlines. The mCherry-LC3 images were processed to enhance local contrast and allow robust thresholding of autophagosomes. Firstly, these images were processed through a Tophat filter (disk, element size=10), to enhance contrast between small speckles (autophagosomes) and cytoplasmic background. The mCherry images were then masked to hide nuclei, which can contain large mCherry-LC3 features^26,27^. Autophagosomes were identified by a per-object thresholding, background method. Intensity was used to distinguish clumped objects. Transfected cells were then further analysed and parameters including location of GFP signal intensity, nuclear size and shape, cell size and shape were calculated (see Table 2 for measurements collected). The image analysis pipeline described is available online, along with supporting documentation (Data Citation 3).

### Data analysis

Data from CellProfiler was output into an SQL database and subsequently imported into the statistical programming environment “R”. Graphs and statistical analyses were generated using automated scripts to obtain, normalise and plot plate data from the entire dataset. In addition as a quality control measure CellProfiler Analyst was used to obtain plate- and image-level views of selected readouts to control for data processing errors and check for systematic bias in the data (edge or plate position effects). In R, data was plotted as boxplots (after Tukey) using ggplot2, and statistical differences determined using the Wilcoxon-Mann-Whitney test (comparing each test well against control mEmerald-only wells from the same plate), followed by a Benjamini & Hochberg *P*-value adjustment to control for multiple comparisons (functions used were wilcox.test and p.adjust, respectively).

### Code availability

The CellProfiler pipeline is publicly available at Harvard Dataverse (Data Citation 3).

## Data Records

### CDS list

A FASTA file of the LF82 CDSs cloned for the library “CDS_LF82_gene_list.fasta” (Data Citation 1).

### Cloning primers

Primer sequences for generation of AIEC CDS clones are given in the file “Library_primers_and_common_primersv2” (Data Citation 2). This also includes the common primers used to add recombination-capable extensions.

### Screening plate layout

The plate layout for all plates used in the screen is given in the document “Layout of library transfections in 60 well format.csv” (Data Citation 4). This is provided in a human and machine-readable CSV format to allow automated annotation of screen data during re-analysis.

### Screening images

The complete set of 27,000 images across four fluorescent channels has been deposited in Harvard Dataverse (Data Citation 5). These are in TIFF format and split by plate as acquired. The data is arranged by plate in zip files named e.g. “Set1_Part1_Rep1”, where Set and Part refers to the clone set (each set is split across two plates (parts)), and Rep the replicate. Original images are similarly named with the addition of e.g. “_B03_s1_w2” where the first part refers to the well address, then site within the well, and finally the imaging channel (w1=DAPI, w2=GFP, w3=mCherry-LC3, w4=actin). Set5 contains a replicate of Set4 with additional controls included (DMSO and Torin).

### Image analysis files

For each set of images there are several data files generated by the CellProfiler pipeline. These are arranged to contain data from different subsets of the analysis (Data Citation 6). Files named _Per_Image contain parameters relating to the entire image field e.g. number of cells, total autophagosome area. _Per_Cell_, _Per_Transfected_Nuclei and _Per_autophagosomes treat each cell outline, nucleus or autophagosome as an object, and present the data related to each object. These are described in Table 2. Object parameters are described in Table 3.

### Cell profiler pipeline

The CellProfiler pipeline is also available (Data Citation 3), along with a flow diagram illustrating the major features and intentions of each step – “CellProfiler_Pipeline_documented.pdf”.

## Technical Validation

### Library validation and plate identification

The identity and sequence of the clones in the library were verified by Sanger sequencing of clones following DNA purification. Incorrectly located or incorrectly-sequenced clones were removed and replaced in the final library plates.

Prior to transfection and imaging, transfection plates were prepared with an internal coding method. Since the top row of wells are not used in the screen, these were filled with cells according to the following formula: plate 1, well A1 filled; plate 2, wells A1 and 2 filled; plate 3, wells A1, 2 and 3 filled. In this fashion data can be matched unambiguously to a plate even if plates are loaded and imaged out-of-order. This links the plate images directly to a plate even in the absence of an external plate barcode reader and barcoded plates.

### Quality control of data collection and image analysis

Each plate contained several negative control wells: untransfected cells, and mEmerald-only transfected cells. In addition, to control for autophagy induction cells transfected with GFP-p62 (known to form ubiquitinated aggregates, be targeted by autophagy and recruit LC3) were included^28^. Cells treated with rapamycin were also included. Plates were assayed in duplicate, with replicates performed on separate days to avoid batch effects.

To control for total variation in cell-level autophagy across the whole screen, mEmerald-transfected negative control wells were pooled together across all plates and are presented as “pooled control data”. This gives an indication of the variability of baseline autophagy in transfected cells across all plates and replicates.

**Figure 2:**
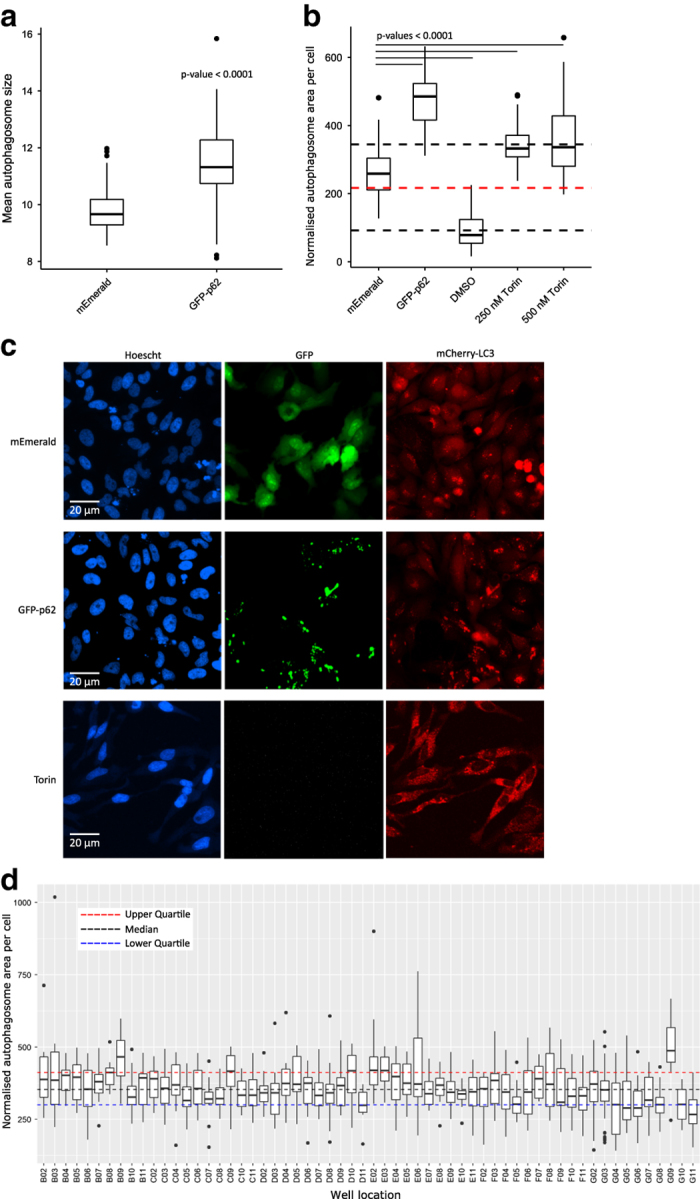
Autophagy induced by overexpression of GFP-p62 or Torin can be robustly detected. (**a**) Quantification of the size of individual autophagosomes per field of view shows larger mCherry-LC3 puncta upon expression of GFP-p62 compared to mEmerald alone. Samples were compared using the Wilcoxon rank sum test, *n*=72 fields of view, representing ~90 transfected cells per view. (**b**) Induction of autophagy by Torin is also robustly detected. In a separate experiment mCherry-LC3 cells were transfected with either mEmerald vector or GFP-p62, or treated with DMSO or Torin. Torin gave a robust level of autophagy induction, with total area of LC3 puncta per cell increasing above those of mEmerald-transfected cells or those treated with DMSO alone. Median (red) and quartiles of the entire dataset are shown with the dotted lines. Samples were compared to mEmerald controls using the Wilcoxon rank sum test, *n*=24 fields of view per condition, representing ~90 cells per view. (**c**) mEmerald vector-transfected cells show low levels of autophagy as indicated by mCherry-LC3 puncta. In contrast, expression of GFP-p62 results in intense GFP-p62 aggregation and co-localisation of GFP-p62 and mCherry-LC3. Treatment of cells with Torin also results in increased numbers of autophagosomes, but of a smaller size to those seen with GFP-p62 (note that the Torin image is from a separate experiment, shown in B above). (**d**) Boxplot representing a single plate (Set2 Part1) showing normalised total autophagosome area per cell. Boxes represent data for each well between 1st and 3rd quartiles, whiskers span points 1.5x the inter-quartile range, and dots represent datapoints outside of this range. The autophagy induction induced by p62 (G09) is marked compared to other proteins. G02 and G03 represent mEmerald controls and are pooled from across the entire plate set (thus representing the total variation within the screen). G11 are untransfected cells. Single-plate-derived median and quartiles are shown with coloured dotted lines.

We found that drug treatment by rapamycin, rapamycin plus ammonium chloride, or ammonium chloride alone, did not induce measurable levels of autophagy. This is similar to what has been previously observed with rapamycin being a weak inducer of autophagy under some conditions^29^, and in many cases other drugs such as Torin are now preferred^30^. However, expression of GFP-p62 induced robust increases in the size of individual LC3 puncta (Fig. 2a), and also showed strong co-localisation with mCherry-LC3–likely representing recruitment of LC3 to p62 aggregates. This positive control was amongst the highest inducer of autophagic puncta observed in the screen (see Fig. 2d), as well as showing the most co-localisation between GFP and mCherry-LC3 signals (Table 4). It is worth noting that co-localisation of GFP and mCherry-LC3 is performed without reference to autophagosome detection by the analysis pipeline, and thus represents an autophagosome-detection independent measure of autophagosome localisation to GFP-fusion proteins.

This indicated that the detection of autophagy induction by our imaging pipeline worked, but other treatments failed to induce sufficient autophagy to be detected by autophagosome number or area methods. To ensure this was not due to sensitivity problems with the screening approach, we performed an additional test using cells treated with Torin, DMSO or transfected GFP-p62. In this test we were able to robustly measure increased total autophagosome area in cells treated with two concentrations of Torin, confirming that the autophagosome detection pipeline functions as intended (Fig. 2b). This image data is recorded in Set5.

### Validation of localisation and morphology measurements

**Figure 3:**
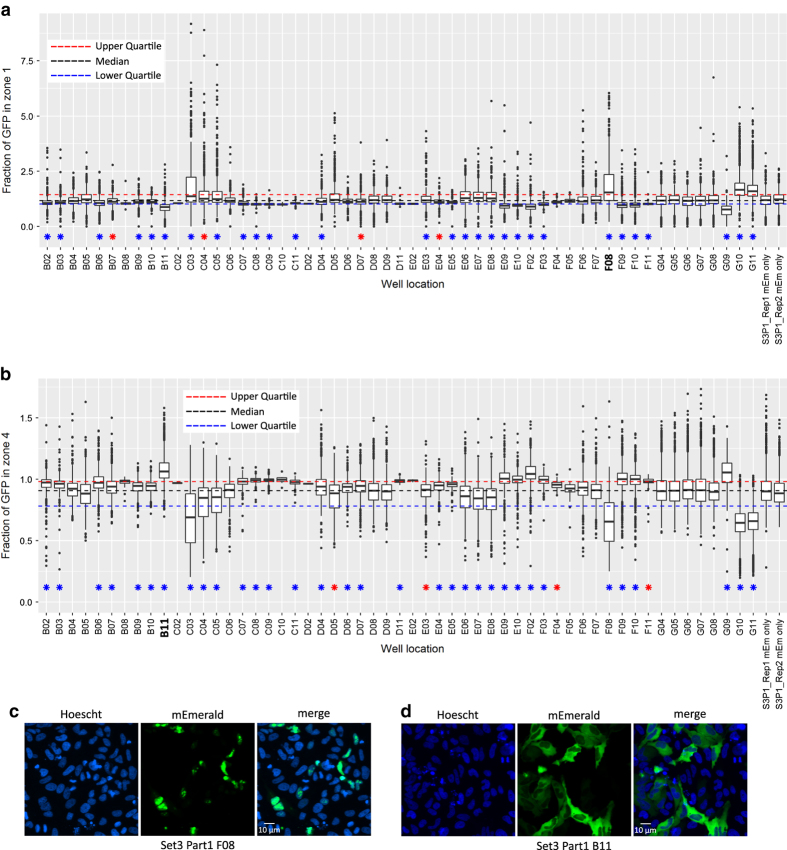
Automated localisation of mEmerald fusions. (**a**) Box and whisker plot showing the fluorescence intensity of mEmerald-protein fusions within radial zone 1 (nucleus) of transfected cells pooled from 2 replicates of a representative plate. Blue and red stars indicate significant differences from control mEmerald cells of *P*<0.01 and *P*<0.05 respectively (Wilcoxon test, Benjamini & Hochberg corrected *P* values). The final 2 bars represent pooled control wells from the two individual replicate plates as a control for inter-plate variation. Note that B11 and F08 represent reduced or increased nuclear localisation, respectively. (**b**) Box and whisker plot showing mEmerald fluorescence intensity of radial zone 4 (cytoplasm) of transfected cells. Images analysed and representation are the same as in A. Note the reciprocal relationship between scores in zones 1 and 4. (**c**) Representative images of well F08, showing nuclear localisation of the mEmerald-LF82_452 fusion protein. (**d**) Representative images of well B11, showing nuclear exclusion and cytoplasmic localisation of mEmerald-LF82_762.

For other readouts such as localisation information and cellular morphology changes, we selected wells of interest based upon examination of boxplots generated from the CellProfiler data output. Wells of interest were notably altered in value distribution to controls, or scored as significantly different by our statistical testing. We then examined sample images manually to ensure that the pipeline had correctly scored these features. Examples of two localisation phenotypes are given in Figs 3c–f.

In one case two clones of the same DNA construct were transfected in the same plates – these score very closely in localisation measurements, e.g. clone LF82_722, set 3, part 1, F9 and E10; median radial zone 4 scores of 1.59 (s.d. 0.21) or 1.58 (s.d. 0.16). In other cases where DNAs were re-transfected as part of set 4 to confirm phenotypes they also scored extremely similarly e.g. LF82_314 with median radial zone 4 scores of 1.67 (s.d. 0.26) (set 1, part 2, E04) or 1.56 (s.d. 0.17) (set 4). A fuller list of radial zone 4 GFP measurements is given in Table 5.

## Usage Notes

Raw images in TIFF format are freely available for re-analysis by any capable image processing software, or for manual inspection. Although acquired with identical imaging settings, some inter-plate normalisation may be required–we would recommend using negative control wells (untransfected and mEmerald-only) to perform data normalisation between plates. Note that Set5 was captured at a different time, following microscope laser replacements, and so absolute intensity values differ from those of the other plates as a result.

Data in CellProfiler-generated analysis files is given as CSV text and can be imported into any appropriate software for re-analysis. These tables can also be correlated with the gene / clone layout records given above to allow retrieval of specific images or image data.

The original CellProfiler pipeline is also available and we would recommend using this with a small test set of images to become familiar with the resulting data files and their relationship to the images before embarking upon large-scale re-analysis, since some of the parameter naming and object vs image details can be confusing at first glance. Alongside the pipeline is a document including a flow-chart outlining the steps in the pipeline and their purpose. Similarly, the CellProfiler pipeline contains notes as to the purpose of each module and step in the workflow that will aid understanding of the resulting data.

## Additional information

**How to cite this article:** Collins, A. & Huett, A. multi-phenotypic imaging screen to identify bacterial effectors by exogenous expression in a HeLa cell line. *Sci. Data* 5:180081 doi: 10.1038/sdata.2018.81 (2018).

**Publisher’s note:** Springer Nature remains neutral with regard to jurisdictional claims in published maps and institutional affiliations.

## Supplementary Material



## Figures and Tables

**Table 1:** Strains used for genome comparison with AIEC LF82.

**Strain name**	**NCBI accession number**
EHEC O111:H	NC_013364.1
EPEC O127:H6	NC_011601.1
UPEC 536	NC_008253.1
K12 DH10B	NC_010473.1
K12 MG1655	NC_000913.3
K12 W3110	NC_007779.1
Genomes from these strains were used to filter the LF82 genome to remove genes common to other pathovars or lab strains of *E. coli*. In this way an LF82-restricted gene library was created and cloned.	

**Table 2:** Image analysis files produced by CellProfiler and subsequently modified for use in data analysis and figure production.

**File Name**	**Description**
MyExpt_Per_AllNuclei.txt	Measurements of identified nuclei
MyExpt_Per_autophagosomes.txt	Measurements of identified autophagosomes
MyExpt_Per_Image.txt	Measurements from each field of view
MyExpt_Per_Transfected_cells.txt	Measurements of all cells identified to contain GFP (successfully transfected cells)
MyExpt_Per_Transfected_cytoplassm.txt	Measurements of cytoplasm associated with transfected cells (cytoplasm defined as the area within the cell outline, excluding the nucleus)
MyExpt_Per_Transfected_Nuclei.txt	Measurements of all nuclei associated with transfected cells
Per_Image_data_with_pooled_mEm_controls.txt	Same as MyExpt_Per_Image but only for wells G02 and G03 (mEmerald-only control wells)
Per_Image_mEm_data_added_control_GFP_data_removed.txt	Same as above, but with data from wells G10 and G11 removed. Data was removed due to the transfected vector not encoding GFP (well G10) or as there was no GFP to detect, as cells were not transfected (well G11).
PerCellData_Cell_Cytoplasm_Nuclei_combined_with_well_and_plate_data_added.txt	Dataset containing individual measurements for all transfected cells, cytoplasm and nuclei. This dataset was used for most data analysis and production of graphs. It is a merged dataset, combining MyExpt_Per_Transfected_cells, MyExpt_Per_Transfected_Cytoplasm, and MyExpt_Per_Transfected_Nuclei.
Note that in some files control data has been removed and analysed separately, before being merged in final combined files. This allowed control wells lacking GFP fluorescence to be correctly analysed in CellProfiler, and also enabled controls to be pooled across plates to demonstrate the consistency of the entire screen.	

**Table 3:** Parameters used by the MeasureObjectSizeShape module in CellProfiler.

**Object measurement field**	**Field description**
AreaShape_Center_X or _Y	Coordinates of the object centroid (point farthest from object edge).
AreaShape_Area	Area of an object in pixels.
AreaShape_Compactness	Mean squared distance of object pixels from centroid, divided by object area.
AreaShape_Eccentricity	Eccentricity of an imaginary best-fitting ellipse encompassing the object.
AreaShape_EulerNumber	Number of objects minus number of holes within objects.
AreaShape_FormFactor	4*π*Area/Perimeter^2^. Equals 1 for a perfectly circular object.
AreaShape_MajorAxisLength AreaShape_MinorAxisLength	Lengths of long or short axes of the ellipse fitted to object.
AreaShape_MaxFeretDiameter AreaShape_MinFeretDiameter	Maximum/minimum distances between two parallel tangents drawn touching the object and rotated around it.
AreaShape_MaximumRadius	The maximum distance of any object pixel to the closest pixel outside of the object.
AreaShape_MeanRadius	Mean distance of an object pixel to the closest pixel outside the object.
AreaShape_Orientation	Angle in degrees between x-axis and major axis of an ellipse that best fits the object.
AreaShape_Perimeter	Total number of pixels forming object perimeter
AreaShape_Solidity	Portion of pixels that are part of the object within the convex hull (minimum shape encompassing entire object). 1=no holes or concave boundary, <1=holes or convex boundary.
These measurements are produced for every object if so designated in the pipeline. They allow individual objects to be used in subsequent analysis, or grouped at a per-cell, per-image, or per-treatment level.	

**Table 4:** Top 20 correlations between GFP and LC3.

**Top 20 correlations between GFP and LC3 in transfected cells**				
**Plate**	**Well name**	**Gene**	**Median correlation**	**Standard deviation**
Set1_Part1	C09	LF82_153	2.93	1.15
Set1_Part1	C08	LF82_152	2.75	1.51
Set3_Part2	F08	LF82_266	2.63	1.03
Set3_Part2	G09	p62	2.61	0.80
Set1_Part1	E05	LF82_326	2.57	1.44
Set3_Part2	C05	LF82_758	2.53	1.26
Set3_Part2	E08	LF82_121	2.50	1.12
Set1_Part1	E06	LF82_327	2.46	1.83
Set1_Part1	D09	LF82_250	2.44	1.61
Set1_Part1	E04	LF82_325	2.40	1.49
Set1_Part1	F11	LF82_603	2.27	1.47
Set1_Part1	B08	LF82_091	2.19	1.54
Set1_Part1	D08	LF82_248	2.13	1.47
Set1_Part1	E09	LF82_355	2.07	1.54
Set1_Part1	F07	LF82_537	2.05	1.50
Set3_Part2	C06	LF82_109	2.04	1.39
Set1_Part1	E07	LF82_350	2.01	1.34
Set3_Part2	D05	LF82_336	1.99	0.89
Set3_Part2	D04	LF82_0556	1.94	1.44
Set1_Part1	F02	LF82_434	1.94	1.53
Set1_Part1	E08	LF82_353	1.94	1.29
As an alternative readout for finding autophagy substrates/triggers we examined GFP/LC3 correlation. Note that p62 appears close to the top of the list, validating the approach (since autophagy adapters are known to be preferred substrates for autophagy, and to recruit LC3 to auto-aggregates).				
To control for variation in transfection efficiency between DNA constructs we designed our analysis pipeline to only measure properties of transfected cells exhibiting GFP fluorescence. In this manner we were able to observe genuine signals induced by bacterial protein expression without being swamped by background noise from untransfected cells. The threshold for selecting cells as transfected was determined from samples taken from across untransfected wells, and was designed to use both a median intensity and mean absolute deviation intensity, to avoid under-selection of cells with either diffuse, or highly punctate signals. By this measure transfection efficiency ranged between 40-60% for LF82 genes, and 60% for empty pCMV-mEm-4GS vector. An average of 183 cells were present per site, with 90 successfully transfected cells for LF82 genes and 112 successfully transfected cells for control well sites.				

**Table 5:** Top 20 proteins with majority of GFP signal within radial zone 4.

**Top 20 fractions of total GFP signal within radial zone 4**				
**Plate**	**Well name**	**Gene**	**Median fraction**	**Standard deviation**
Set3_Part1	B11	LF82_468	1.69	0.16
Set4	F03	LF82_314	1.67	0.26
Set3_Part1	G09	p62	1.66	0.27
Set3_Part1	F02	LF82_1175	1.63	0.20
Set2_Part1	D11	LF82_259	1.61	0.18
Set3_Part2	F08	LF82_266	1.61	0.04
Set3_Part2	E08	LF82_121	1.60	0.05
Set3_Part1	E09	LF82_004	1.60	0.20
Set3_Part1	C10	LF82_441	1.59	0.04
Set3_Part2	D06	LF82_347	1.59	0.18
Set3_Part1	F03	LF82_539	1.59	0.09
Set3_Part1	F09	LF82_772	1.59	0.21
Set3_Part1	E10	LF82_772	1.58	0.16
Set3_Part1	F10	LF82_793	1.58	0.15
Set4	C02	LF82_788	1.58	0.12
Set3_Part2	C05	LF82_758	1.58	0.17
Set3_Part1	C09	LF82_394	1.57	0.04
Set3_Part1	C08	LF82_356	1.57	0.06
Set4	E02	LF82_759	1.57	0.07
Set2_Part1	F10	LF82_0557	1.57	0.12
Set1_Part2	E04	LF82_314	1.56	0.17
Radial zone 4 represents the outermost portion of the cell, thus these proteins localise to the cytoplasm, plasma membrane or other compartments associated with cell edges. Note that LF82_314 appears twice, due to being replicated between two sets of plates (Set 1 and Set 4). LF82_772 also appears twice as it is replicated in neighbouring wells of the same plates. p62 scores very highly – unsurprisingly given its known cytoplasmic localisation.				
